# Identification of somatic mutations using whole-exome sequencing in Korean patients with acute myeloid leukemia

**DOI:** 10.1186/s12881-017-0382-y

**Published:** 2017-03-01

**Authors:** Seong Gu Heo, Youngil Koh, Jong Kwang Kim, Jongsun Jung, Hyung-Lae Kim, Sung-Soo Yoon, Ji Wan Park

**Affiliations:** 1 Department of Medical Genetics, College of Medicine, Hallym University, 1 Hallymdaehak-gil, Chuncheon, Gangwon-do 24252 Republic of Korea; 2 Wide River Institute of Immunology, Seoul National University, Hongcheon, Republic of Korea; 3 Department of Internal Medicine, Seoul National University Hospital, Seoul, Republic of Korea; 4 Omics Core Lab., National Cancer Center, Goyang, Republic of Korea; 5 The Catholic University, Seoul, Republic of Korea; 6 Syntekabio Inc., Seoul, Republic of Korea; 7 Department of Biochemistry, School of Medicine, Ewha Womans University, Seoul, Republic of Korea

**Keywords:** Acute myeloid leukemia, Gene ontology, Pathway analysis, Somatic mutation, Subtype-specific mutation, Whole-exome sequencing

## Abstract

**Background:**

Acute myeloid leukemia (AML) is a biologically and clinically heterogeneous cancer of the bone marrow that is characterized by the rapid growth of abnormal myeloid cells.

**Methods:**

We performed a mutational analysis to identify AML somatic mutations using the whole-exome sequencing data of 36 tumor-normal sample pairs from Korean patients with *de novo* AML. We explored the functional impact of the genes identified in the mutational analyses through an integrated Gene Ontology (GO) and pathway analysis.

**Results:**

A total of 11 genes, including *NEFH* (*p* = 6.27 × 10^−13^ and *q* = 1.18 × 10^−8^) and *TMPRSS13* (*p* = 1.40 × 10^−10^ and *q* = 1.32 × 10^−6^), also demonstrated *q* values less than 0.1 in 36 Korean AML patients. Five out of the 11 novel genes have previously been reported to be associated with other cancers. Two gene mutations, *CEBPA* (*p* = 5.22 × 10^−5^) and *ATXN3* (*p* = 9.75 × 10^−4^), showed statistical significance exclusively in the M2 and M3 subtypes of the French-American-British classifications, respectively. A total of 501 genes harbored 478 missense, 22 nonsense, 93 frameshift indels, and/or three stop codon deletions and these gene mutations significantly enriched GO terms for signal transduction (GO:0007165, *p* = 1.77 × 10^−3^), plasma membrane (GO:0005886, *p* = 3.07 × 10^−4^), and scaffold protein binding (GO:0097110, *p* = 8.65 × 10^−4^). The mitogen-activated protein kinase (hsa04010, 7.67 × 10^−4^) was the most enriched Kyoto Encyclopedia of Genes and Genomes pathway.

**Conclusions:**

Morphological AML subtypes may in part reflect subtype specific patterns of genomic alterations. Following validation, future studies to evaluate the usefulness of these genes in genetic testing for the early diagnosis and prognostic prediction of AML patients would be worthwhile.

**Electronic supplementary material:**

The online version of this article (doi:10.1186/s12881-017-0382-y) contains supplementary material, which is available to authorized users.

## Background

Acute myeloid leukemia (AML) is a highly malignant cancer of the bone marrow that is characterized by the rapid growth of abnormal myeloid cells. In 2012, leukemia accounted for 2.5% and 3.2% of all new cancer cases and deaths worldwide, respectively [1]. The incidence rate of leukemia increased from 4.7 to 5 cases per 10,000 Koreans from 1999–2010 [2]. AML cytogenetic studies provide important diagnostic and prognostic information for AML patients. However, approximately 50% of AML patients have a normal karyotype (NK-AML). Although Schlenck et al. showed that the combination of the mutations in *FLT* and *NPM1* or CCAAT/enhancer binding protein (C/EBP), alpha (*CEBPA*) could be used to predict NK-AML prognosis, most patients did not have this mutation set [3]. This finding suggests that AML is a highly heterogeneous disease and that a large number of causal mutations have not yet been uncovered [4]. Two systems, the French-American-British (FAB) classification and the newer World Health Organization (WHO) classification, have been used to classify AML into subtypes. To compare our results with the previous studies, we used the FAB system, the most commonly used classification in previous sequencing studies for AML, including The Cancer Genome Atlas (TCGA) whole genome sequencing (WGS) [5, 6]. AML is classified into eight subtypes (M0 through M7) according to the FAB classification based on its morphological features: early forms in white blood cells (M0-M5), red blood cells (M6), and platelets (M7) [3].

Next-generation sequencing technologies have extended AML genetic studies to a genome-wide scope at a single-base resolution. Ley et al. (2008) performed the first AML WGS study on one Caucasian woman with the cytogenetically normal AML subtype M1 and reported non-synonymous single nucleotide variants (nsSNVs) in eight genes (i.e., *CDH24*, *PCLKC*, *GPR123*, *EBI2*, *PTPRT*, *KNDC1*, *SLC15A1*, and *GRINL1B*) and insertions in the coding regions of the *FLT3* and *NPM1* genes [7]. Mardis and his colleagues reported that 16% of 80 NK-AML patients had a somatic point mutation in the *IDH1* gene [8]. According to a later report by Ley *et al*. (2010), approximately 22% of 281 AML patients had *DNMT3A* mutations that were newly discovered using targeted sequencing [9]. In a Chinese study using targeted exome sequencing, the patients with the *DNMT3A* Arg882 mutation showed poor prognosis among AML-M5 individuals [10]. A WGS study with eight Caucasian AML patients showed clonal evolution patterns and mutations associated with relapsed AML in the genes *WAC*, *SMC3*, *DIS3*, *DDX41*, and *DAXX* [11]. Recently, TCGA analyzed 50 and 150 patients with *de novo* AML using WGS and whole-exome sequencing (WES), respectively. They found 23 significantly mutated genes and 237 gene mutations that recurred in at least two patients, which were grouped into nine categories according to their biological functions [6].

AML is a clinically and genetically heterogeneous disease, hence discovering subtype-specific mutations may provide additional prognostic information for AML patients. In this study, we aimed to replicate previous findings in the European studies and to characterize the landscape of somatic mutations present in Korean acute myeloid leukemia. We also performed a stratified analysis for FAB M2- and M3-subtypes to investigate if certain mutations have subtype-specific effects. We subsequently evaluated the functional properties of the significantly mutated genes using an integrated systems analysis of Gene Ontology (GO) and biological pathways.

## Methods

### Patients and samples

We included 36 Korean patients with *de novo* AML who visited the Division of Hematology and Medical Oncology, Seoul National University Hospital, from 1995 to 2013 who had not received a bone marrow transplant prior to sampling. All subjects provided matched tumor-normal sample pairs that passed a DNA quality control (QC) test. The Institutional Review Board for Human Research at Seoul National University approved the study protocol (IRB number 1201-099-396), and all participants signed informed consent forms for WES. A clinician conducted a retrospective medical record review to obtain clinical data including disease status and blood chemistry. An approximately 10-mL bone marrow sample was aspirated from each participant by a clinician in an aseptic environment, and genomic DNA was isolated using the QIAamp DNA Blood Maxi Kit following the manufacturer’s instructions (Qiagen, *Inc.*, Valencia, CA, USA). A 2-ml whole-saliva sample was obtained from the same individuals for the matched normal samples, and the genomic DNA was extracted using the Oragen DNA Self-Collection kit (DNA Genotek, *Inc*., Ontario, Canada).

### Whole-exome sequencing

We captured the target DNA sample using the Agilent SureSelect Human All Exon 50 Mb Kit (Agilent Technologies *Inc.*, Santa Clara, CA, USA). The entire exome regions for both the tumor and normal samples from 36 AML patients were sequenced using the HiSeq 2000 platform with a 100 bp paired-end read protocol (Illumina, *Inc*. San Diego, CA, USA). Each the tumor and the normal sample were sequenced to an average read depth of 76X. The fastq quality score and read length cutoff determined by the NGS QC-toolkit were set to 20 and 70, respectively [12]. We aligned the filtered reads to the reference assembly of human_g1k_v37 fasta using BWA-0.7.5 [13] and called somatic SNVs using MuTect v1.1.4 according to the Catalogue of Somatic Mutations in Cancer (COSMIC) v68 database [14]. We used Varscan v2.3.6 to call short indels [15]. We obtained translational effect of variant allele, protein change information, mutation reports in Catalog of Somatic Mutations in Cancer, and predictions of coding non-synonymous variants on protein function using the Oncotator web application [16]. Preliminary reports using six of 36 samples have been previously published elsewhere (Additional file 1: Table S1) [17–19].

### Statistical analysis

We performed a mutation significance test using MutSigCV v1.4 with a significance threshold of a *p* value less than 0.05. The *p* value was calculated with the chi-square test to determine whether the observed mutations in a gene significantly exceeded the expected frequency of random background mutations. We additionally considered a *q* value less than 0.1, which was an analogue of the *p* value calculated based on the Benjamini-Hochberg false discovery rate (FDR) [20]. To identify subtype-specific mutant genes, we performed the test individually in each of the subgroups as follows: M2-AML, acute myeloblastic leukemia with maturation, and M3-AML, acute promyelocytic leukemia. We screened for mutations that recurred in more than two patients with a *p* value less than 0.05 in any patient group. We categorized a mutant gene as a subtype-specific gene if the statistical significance of any one subgroup (i.e., M2 or M3) represented exclusively the significance of the total patient group. Finally, we systematically searched the PubMed database (www.ncbi.nlm.nih.gov/pubmed) to review previous studies on the relevance of the genes with *p* values less than 0.05 and *q* values less than 0.1 for AML and/or other cancers.

### Gene set enrichment analysis and pathway analysis

To investigate the biological relevance of the mutations, we performed a GO enrichment analysis with the Database for Annotation, Visualization, and Integrated Discovery (DAVID 6.8 beta) [21]. We categorized the function of these genes into three classes: ‘biological process’, ‘cellular components’, and ‘molecular function’. We also used the Kyoto Encyclopedia of Genes and Genomes (KEGG) database (http://www.genome.jp/kegg/pathway.html) to identify pathways for the genes that were frequently mutated in AML cells [22].

## Results

The mean age of the 36 AML patients was approximately 46 years. The mean percentage of blasts in the bone marrow (60.93%) in patients in this study was approximately 12-fold higher than the level in patients undergoing complete remission. Overall, the abnormally high WBC count and low platelet count observed in the study subjects represent typical characteristics of AML patients. A total of 32 patients were grouped into five AML subtypes as follows: M1 (*n* = 43), M2 (*n* = 11), M3 (*n* = 12), M4 (*n* = 3), and M5 (*n* = 3). However, four patients were not placed into any of the AML subgroups (Table 1). Details of the 36 AML patients including sex, age, FAB subtype, and cytogenetic abnormalities were shown in Additional file 1: Table S1. Ten of 11 patients with the acute promyelocytic leukemia (APL)-specific chromosomal translocations t(15;17) (q22;q21) were classified into the FAB subtype M3. Eight of 12 AML patients with normal karyotype fell into the M2 subtype.

**Table 1:** Characteristics of the 36 acute myeloid leukemia patients

Variables	AML (*n* = 36)
Age, mean (SE)	46.43 (3.09)
Male (%)	22 (61.11)
Absolute neutrophil count × 10^3^/mm^3^, mean (SE)	1.99 (0.92)
Bone marrow blast (%), mean (SE)	60.93 (4.59)
White blood cell count × 10^3^/mm^3^, Mean (SE)	25.72 (4.99)
Platelet count × 10^3^/mm^3^, Mean (SE)	73.34 (13.03)
Overall survival time (months), mean (SE)	76.84 (31.08)
French-American-British^a^, n	
M1	3
M2	11
M3	12
M4	3
M5	3
Others^b^	4

*Abbreviation*: *AM*L acute myeloid leukemia, *SE* standard error

^a^AML is classified into five subtypes (M1 through M5) according to the French-American-British (FAB) classification

^b^Others are comprised of myelodysplastic syndrome (*n* = 3) or whose subtype information was not available (*n* = 1)

### Mutation detection

We identified a total of 4,954 intragenic somatic mutations and 43 genes passed a mutation significance *p* value threshold 0.01 (data not shown). Among them, 11 genes demonstrated *q* values less than 0.1 in 36 AML patients (Table 2). The most significantly mutated gene in AML cells was the heavy polypeptide gene (*NEFH*, *p* = 6.27 × 10^−13^, *q* = 1.18 × 10^−8^). Frameshift indels potentially lead to the disease since they alter protein amino acid sequences which were found in olfactory receptor family 2 subfamily T member 35 (*OR2T35*) and proprotein convertase subtilisin/kexin type 5 (*PCSK5*). Missense SNVs were found in transmembrane protease, serine 13 gene (*TMPRSS13*) in six samples, keratin associated protein 4–5 (*KRTAP4-5*) in one sample, and G protein regulated inducer of neurite outgrowth 1(*GPRIN1*) in one sample (Table 2 and Additional file 1: Table S2).

**Table 2:** Genes identified through mutational analysis in AML in the total, M2, and M3 subtype patient groups

Gene	Chr	Type of variant (patients, n)	*p* ^a^	*q* ^a^	Previous studies^b^
AML (*p* < 0.01 and *q* < 0.10)					
* NEFH*	22	In Frame Ins(6)/Del(4)	6.27 × 10^-13c^	1.18 × 10^−8^	RCC [23], ESCC [24]
* TMPRSS13*	11	In Frame Del(6), Missense(5)	1.40 × 10^-10c^	1.32 × 10^−6^	
* KRTAP4-5*	17	In Frame Ins(5), Missense(1)	5.07 × 10^-8c^	3.19 × 10^−4^	
* OR2T35*	1	Frame Shift Ins(1)/Del(4)	1.50 × 10^-7c^	7.09 × 10^−4^	
* HAVCR1*	5	Intron SNV(1), In Frame Del(5), Silent(1)	3.62 × 10^-7c^	1.37 × 10^−3^	Colorectal [25], RCC [26]
* IFI27*	14	In Frame Ins(3)	6.88 × 10^-6c^	0.02	Ovarian [27], Breast [28]
* PCSK5*	9	Intron SNV(1), Frame Shift Del(6), 3′UTR Ins(7)/Del(1)	8.50 × 10^-6c^	0.02	Lung [29]
* GPRIN1*	5	Missense(1), In Frame Del(5)	8.88 × 10^-6c^	0.02	
* MRPL18*	6	In Frame Del(3)	2.09 × 10^−5^	0.04	
* ARSD*	X	Intron Ins(2)/Del(2), In Frame Del(4)	2.93 × 10^−5^	0.05	CLL [30]
* MAML3*	4	In Frame Ins(2)/Del(4), Silent(3), Intron Del(1)	3.33 × 10^−5^	0.05	
AML-M2 (*p* < 0.01)					
* CEBPA*	19	In Frame Ins(3)	5.22 × 10^−5^	0.49	HCC [31], AML [32]
* EP400*	12	Intron Del(1), In Frame Ins(4)/Del(1)	3.45 × 10^−4^	1	RCC [33], Colorectal [34]
AML-M3 (*p* < 0.01)					
* ATXN3*	14	In Frame Ins(2), Intron Ins(1)	9.75 × 10^−4^	1	Lung [35]

*Abbreviation*: *AML* acute myeloid leukemia, *B-CLL* B-cell chronic lymphocytic leukemia, *Breast* breast cancer, *Colorectal* colorectal cancer, *CLL* chronic lymphocytic leukemia, *Chr* chromosome, *Del* deletion, *ESCC* esophageal squamous cell carcinoma, *HCC* hepatocellular carcinoma, *Ins* insertion, *Lung* lung cancer, *MDS* myelodysplastic syndrome, *NMSC* non-melanoma skin cancer, *Ovarian* ovarian cancer, *RCC* renal cell carcinoma, *SNV* single nucleotide variant

^a^ *p* and *q* values were obtained from mutational analysis

^b^Cancers reported by previous studies to have associations with the gene mutations

^c^The genes still being significant after Bonferroni correction

### Subtype-specific somatic mutations

**Fig. 1:**
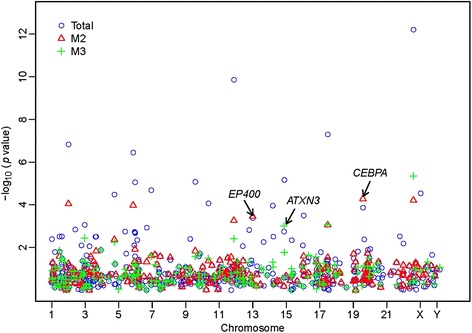
Three genes demonstrating AML subtype specificity (*p* < 0.01). The symbols, circle (○), triangle (△), and cross (+) denote the significance levels of each gene estimated in the total, M2- and M3-subtype groups, respectively. The labeled genes demonstrate that their mutational significance levels are higher in a subtype group than in the total group

We identified three significantly mutated genes with a *p*-value of less than 0.01 for each M2 and M3 group. Among them, we identified two genes, *CEBPA* (19q13.1, *p* = 5.22 × 10^−5^) and E1A binding protein p400 (*EP400*, 12q24.33, *p* = 4.45 × 10^−4^), that showed statistical significance exclusively in the M2 subtype, as well as one gene, ataxin 3 (*ATXN3*, *p* = 9.75 × 10^−4^) that was exclusive for the M3 subtype. In other words, these mutations were found to be more frequent in either M2 or M3 subgroup cases than in the total cohort of 36 AML patients (Table 2). Although only eight genes identified in all cases after Bonferroni correction for multiple testing (with a *p* value cutoff of 1 × 10^−5^ for the 4,954 genes under test), 10 among the 16 genes listed in Table 2 were cancer-relevant genes reported by previous studies. Especially, early studies revealed an association between the *CEBPA* mutations and AML. Figure 1 summarizes the results of our mutational analysis. The –log10 of the *p* value for each mutation (y-axis) was plotted against their respective chromosomal position (x-axis). Specifically, the three subtype-specific mutant genes with a *p* value of less than 0.01were labelled in the plot.

### Pathways and biological processes

We identified total of 501 genes harbored 478 missense, 22 nonsense, 93 frameshift indels, and/or three stop codon deletions. We performed GO and KEGG pathway enrichment analyses with the input consisted of the 501 genes. The GO analysis demonstrated that 43 mutated genes could contribute to AML tumorigenesis through the biological processes such as signal transduction (*p* = 1.77 × 10^−3^, Fold Enrichment = 1.63). In terms of cellular components, the plasma membrane showed the most significant alteration (*p* = 3.07 × 10^−4^, Fold Enrichment = 1.32). Scaffold protein binding was the most significantly altered molecular function (*p* = 8.65 × 10^−4^, Fold Enrichment = 6.18). To investigate the pathway-level relationship of the mutated genes, we performed a pathway analysis in the KEGG database (Additional file 1: Table S3). Mitogen-activated protein kinase signaling (*p* = 7.67 × 10^−4^, Fold Enrichment = 2.50) and long-term potentiation (*p* = 2.40 × 10^−3^, Fold Enrichment = 4.28) pathways were significantly altered in AML.

## Discussion

A total of 11 genes were significantly mutated in 36 AML patients (*p* < 0.01 and *q* < 0.1). Specifically, *NEFH*, hepatitis A virus cellular receptor 1 (*HAVCR1*, 5q33.2), interferon, alpha-inducible protein 27 (*IFI27*, 14q32), *PCSK5*, and the arylsulfatase D gene (*ARSD*, Xp22.3) have been implicated in a variety of cancers. For instance, the variants of *NEFH* gene, a tumor suppressor, were suggested as prognostic markers for renal cell carcinoma (RCC) and contributed to susceptibility of esophageal squamous cell and hepatocellular carcinomas [24, 36]. While overexpression of this gene interrupts the development of cell structure and function in normal cells [37], loss-of-function mutations in this gene activate the Akt/β-catenin pathway and cause increased glycolysis and result in mitochondrial dysfunction in cancer cells [24]. The transmembrane protease, serine 13 gene (*TMPRSS13*, 11q23), a splice variant of mosaic serine protease large form (MSPL), encodes a family of the type II transmembrane serine proteases which plays critical roles in maintaining homeostasis, infection, and tumorigenesis [38, 39]. The *HAVCR1* gene is a biomarker for diagnosing renal cell, ovarian, and colorectal carcinoma [25, 40]. Especially, elevated expression of this gene prevents cancer cell invasion and adhesion in colorectal cancer cells [25]. *IFI27*, the most highly up-regulated gene in human whole blood, is related with immune response through activation of T lymphocytes and dendritic cells [41]. Furthermore, this gene induces the interferon-alpha and stimulates myeloid dendritic cells [42]. The proprotein convertases (PCs) play important roles in development and metastasis of multiple cancers. The *PCSK5* gene (also known as PC5 or PC6) has been reported to be systematically down-regulated in intestinal tumors of the knockout mouse model and human [43].

The protein encoded by the *ARSD* gene located on X chromosome is a member of the sulfatase family, which is an essential element for skeletal and cartilage growth. The elevated expression of this protein was suggested to be associated with lipid metabolism such as sphingolipid and development of chronic lymphocytic leukemia [30]. A cluster of Mastermind-like (MAML) genes, including *MAML1*, *MAML2*, and *MAML3*, encodes transcriptional co-activators for various signal pathways such as Notch signaling and tumor suppressor pathway activated in multiple cancers [44]. Specifically, the *MAML3* gene regulates the retinoic acid gene, which inhibits growth of human tumor cells [45]. We identified four novel genes, *KRTAP4-5*, *OR2T35*, *GPRIN1*, and *MRPL18,* of unknown function in tumor progression and metastasis.

The genes mutated exclusively in the patients with the AML subtypes M2 and M3 have been reported more frequently in previous studies to have an association with AML and/or other types of cancers than the genes identified in the total AML patient group. For instance, previous NGS studies for AML evaluated the prognostic impact of the gene *CEBPA* in AML patients [6, 32]. This gene had the lowest *p* value in the AML M2 group in the current study. The high proportion of M2-patients in the study subjects may have affected the results of previous studies. The E1A binding protein p400 (*EP400*, 12q24.33) was reported to be associated with cancers such as RCC and colorectal cancer. All three genes specific to the M3 subtype (*ATXN3*, thymine-DNA glycosylase (*TDG*, 12q24.1), and *HCLS*) also showed possible associations with cancer risks, such as B-cell chronic lymphocytic leukemia (B-CLL).

Gene set enrichment analysis showed that MAPK signaling pathway was significantly enriched in the 36 Korean AMLs. MAPKs play important role in converting extracellular stimuli into cellular responses and are often altered in cancers [46]. The genomic region, Chromosome 11:117789342–117789345, harbor five missense mutations leading to protein changes, p.A77G and p.Q78R, of transmembrane serine proteases that is known to function in tumorigenesis [39]. We additionally investigated the clinical features of patients who share the same recurrent mutations, including age, absolute neutrophil count, bone marrow blast percentage, white blood cell count, platelet count, and overall survival time, however there were no specific clinical features observed among them.

## Conclusions

In this study, we replicated multiple gene mutations reported by previous European studies in Korean patients. We also discovered novel genes significantly mutated in AMLs and some mutated genes that showed subtype-specific patterns of mutations. The effects of novel genes and subtype-specific somatic mutations in AML warrant further validation in larger cohorts. Following validation, it would be worthwhile in future studies to evaluate the usefulness of these genes in genetic testing for the early diagnosis and prediction of the prognosis of AML patients.

## Additional file

Additional file 1: Table S1. Details of the 36 AML patients. **Table S2.** Functional information for 15 significantly mutated genes in 36 Korean AML patients. **Table S3.** Results of gene ontology and KEGG pathway analyses. (DOCX 38 kb)

## Abbreviations

AML: Acute myeloid leukemia
ANK-AML: AML with an abnormal karyotype
APL: Acute promyelocytic leukemia
CPH: Cox proportional hazards regression
FAB: French-American-British classification
FDR: False discovery rate
GO: Gene ontology
IGV: Integrative genomics viewer
INO80: INO80 complex subunit
JMML: Juvenile myelomonocytic leukemia
LR: Logistic regression
MAPK: Mitogen-activated protein kinase
NGS: Next-generation sequencing technologies
NK-AML: AML with a normal karyotype
nsSNVs: Non-synonymous single nucleotide variants
OR: Odds ratio
TCGA: The Cancer Genome Atlas Research Network
WBC: White blood cells
WES: Whole-exome sequencing
WGS: Whole genome sequencing
