# Supplementary material for: Identification of somatic mutations using whole-exome sequencing in Korean patients with acute myeloid leukemia
Source: BMC Med Genet. 2017 Mar 1;18:23. doi: 10.1186/s12881-017-0382-y (PMC5333433; doi:10.1186/s12881-017-0382-y)
Supplement: Additional file 1: Table S1. — Details of the 36 AML patients. Table S2. Functional information for 15 significantly mutated genes in 36 Korean AML patients. Table S3. Results of gene ontology and KEGG pathway analyses. (DOCX 38 kb) [file 12881_2017_382_MOESM1_ESM.docx]

**Additional file 1**

**Table S1**. Details of the 36 AML patients

| **Sample ID** | **Sex** | **Age** | **ANC**  **(n/mm^3^)** | **Blast (%)** | **WBC**  **(n/mm^3^)** | **PLT**  **(n×10^3^/mm^3^)** | **OS (months)** | **FAB** | **Cytogenetic Abnormality** |
| --- | --- | --- | --- | --- | --- | --- | --- | --- | --- |
| SNUH_G01^a^ | Female | 53 | 2063 | 83.30 | 20630 | 120 | 35.87 | M5 | 46,XX[20]^b^ |
| SNUH_G03^a^ | Male | 53 |  | 79.3 | 6600 | 20 | 37.43 | M2 | 45,X,-Y,t(8;21)(q22;q22)[21] |
| SNUH_G04^a^ | Male | 51 | 25578 | 90.80 | 30300 | 68 | 35.37 | M3 | 46,XY,t(15;17)(q22;q21),inc[2]/46,XY[21] |
| SNUH_G05^a^ | Male | 21 |  | 26.20 | 85200 | 35 | 29.00 | M5b | 46,XY,inv(16)(p13q22)[13]/46,idem,?del(7)(q36)[7] |
| SNUH_G06^a^ | Female | 60 | 695 | 98.20 | 13900 | 58 | 38.17 | M1 | 46~48,XX,+8,+1~2mar,inc[cp11] |
| SNUH_G09^a^ | Female | 45 | 1005 | 45.90 | 7730 | 113 | 40.27 | M2 | 46,XX[20] |
| SNUH_G11 | Male | 30 |  | 70.00 | 7560 | 11 | 207.93 | M4 | 46,XY,t(8;21)(q22;q22)[8]/45,X,-Y,t(8;21)(q22;q22)[3]/45,X,-Y,t(8;21)(q22;q22),add(22)(qter)[9] |
| SNUH_G12 | Female | 59 | 2506 | 28.30 | 7160 | 61 | 53.27 | M2 | 46,XX[20] |
| SNUH_G14 | Male | 30 | 207 | 93.60 | 800 | 27 | 30.00 | M3 | 46,XY,t(15;17)(q22;q21)[20] |
| SNUH_G19 | Male | 68 | 403 | 90.70 | 40300 | 119 | 26.97 | M2 | 46,XY[20] |
| SNUH_G20 | Female | 22 | 1436 | 42.70 | 10260 | 32 | 23.17 | M2 | 46,XX[20] |
| SNUH_G22 | Female | 46 |  |  |  |  | 174.50 | M3 | NA |
| SNUH_G23 | Male | 17 | 485 | 14.80 | 48460 | 84 | 80.43 | M3 | 46,XY,t(15;17)(q22;q21)[27] |
| SNUH_G24 | Male | 17 |  | 61.5 | 17820 | 38 | 105.53 | M4 | t(8;21) |
| SNUH_G25 | Male | 66 | 1035 | 95.10 | 51730 | 32 | 15.97 | M3 | 45,X,-Y,t(15;17)(q22;q21)[20] |
| SNUH_G28 | Female | 62 |  | 29.8 | 7710 | 17 | 12.30 | M2 | 46,XX[20] |
| SNUH_G29 | Female | 54 | 0 | 97.00 | 141500 | 15 | 6.47 | M5 | 46,XX,1qh+,t(11;19)(q23;p13.1)[19]/46,XX,1qh+[1] |
| SNUH_G30 | Male | 71 | 1896 | 76.10 | 23700 | 114 | 24.50 | M2 | 46,XY,del(20)(q11.2q13.2)[2]/46,XY[19] |
| SNUH_G31 | Female | 23 | 0 | 99.00 | 79460 | 25 | 24.43 | M3 | 46,XX[2] |
| SNUH_G33 | Male | 51 | 1299 | 36.10 | 16240 | 94 | 41.10 | M3 | 46,XY,t(15;17)(q22;q21)[18]/46,XY[2] |
| SNUH_G34 | Female | 43 |  | 30.20 | 10000 | 34 | 26.20 | M1 | 46,XX,t(15;17)(q22;q21)[20]/46,XX[1] |
| SNUH_G35 | Male | 62 |  | 80.3 | 36100 | 119 | 85.93 | M4 | No mitosis |
| SNUH_G38 | Male | 67 | 4100 |  | 8200 | 439 | 12.47 | MDS-AML | 46, XY[20] |
| SNUH_G40 | Male | 53 | 1432 | 67.60 | 10230 | 53 | 1134.03 | M3 | 46,XY,t(15;17)(q22;q21)[7]/46,XY[3] |
| SNUH_G48 | Female | 56 |  | 94.5 | 26210 | 59 | 106.83 | M1 | 46,XX[17] |
| SNUH_G49 | Male | 29 | 188 | 51.90 | 6250 | 50 | 95.43 | M3 | 46,XY,t(15;17)(q22;q21)[11]/46,XY[4] |
| SNUH_G51 | Male | 69 | 495 | 9.70 | 1980 | 82 | 22.80 | M3 | 46,XY,t(15;17)(q22;q21)[20]/46,XY[1] |
| SNUH_G54 | Male | 27 | 0 | 36.30 | 1260 | 41 | 38.93 | MDS-AML | 46,XY,del(11)(p13p15)[19]/46,XY[1] |
| SNUH_G61 | Male | 61 | 2493 | 83.5 | 35620 | 111 | 41.57 | M2 | 46,XY[20] |
| SNUH_G62 | Male | 62 | 195 | 55.00 | 1300 | 214 | 26.30 | M3 | 46,XY,t(15;17)(q22;q21),add(21)(q22)[12]/46,XY[8] |
| SNUH_G65 | Female | 76 | 325 | 24.60 | 2500 | 132 | 15.90 | M2 | 47,XX,+4[10]/46,XX[10] |
| SNUH_G68 | Male | 55 | 1167 | 51.90 | 38910 | 63 | 23.37 | M2 | 46,XY[8] |
| SNUH_G76 | Female | 19 | 2648 | 45.50 | 44130 | 8 | 20.67 | M2 | 46,XX[20] |
| SNUH_G78 | Male | 23 | 46 | 90.20 | 460 | 41 | 28.40 | M3 | 46,XY,t(15;17)(q22;q21)[1] |
| SNUH_G80 | Male | 39 | 1474 | 44.6 | 1820 | 27 | 12.23 | MDS-AML | 44~46,XY,add(1)(q21)x2,+der(1;5)(q10;p10),add(4)(q25),+6,der(7)t(7;15)(q11.2;q15),+8,+10,-11,del(12)(p13),-14,-15,-16,-20,+21,+mar1[cp10]/53~58,XY,+X,+der(1;5)(q10;p10),+5,+7,add(7)(q11.2)x2,+8,+8,+9,+10,+13,+15,+21,+22,+22,+mar2[cp14] |
| SNUH_G82 | Male | 59 | 584 | 47.70 | 58370 | 11 | 32.60 | NA | 46,XX,der(16)?inv(16)(p13.1q12)inv(16)(p13.1q22)[15] |

Abbreviation: ANC, absolute neutrophil count; Blast, bone marrow blast; FAB, French-American-British classification; MDS-AML, myelodysplastic syndromes-acute myeloid leukemia; PLT, platelet count; OS, overall survival time; SNUH, Seoul National University Hospital; WBC, white blood cell count.

^a^Samples were previously published elsewhere in preliminary form [17-19]

^b^Numbers in brackets indicate numbers of cells studied.

**Table S2**. Functional information for15 significantly mutated genes in 36 Korean AML patients

| **Gene** | **Chr** | **Start (bp)** | **End (bp)** | **Classification Type** | **Rec^a^** | **REF\|ALT** | **Protein Change** | **COSMIC (n)^b^** | **SIFT (score)^c^** |
| --- | --- | --- | --- | --- | --- | --- | --- | --- | --- |
| **In All** | | | | | | | | | |
| *NEFH* | 22 | 29885567 | 29885568 | In_Frame_Ins | 5 | .\|AAGTCCCCTGAGAAGGCC | p.647_647K>KSPEKAK |  |  |
|  |  | 29885575 | 29885576 | In_Frame_Ins | 1 | .\|TGAGAAGGCCAAGTCCCC | p.649_650PE>PEKAKSPE |  |  |
|  |  | 29885581 | 29885604 | In_Frame_Del | 1 | AGGCCAAGTCCCCAGAGAAGGAAG\|. | p.AKSPEKEE652del |  |  |
|  |  | 29885599 | 29885604 | In_Frame_Del | 3 | AGGAAG\|. | p.EE658del |  |  |
| *TMPRSS13* | 11 | 117789313 | 117789327 | In_Frame_Del | 6 | GGGCTGGAGATGCCT\|. | p.QASPA83del | urinary_tract(1) |  |
|  |  | 117789342 | 117789342 | Missense | 1 | T\|C | p.Q78R |  | T(0.66) |
|  |  | 117789345 | 117789345 | Missense | 4 | G\|C | p.A77G |  | T(0.44) |
| *KRTAP4-5* | 17 | 39305775 | 39305776 | In_Frame_Ins | 5 | .\|GGCAGCAGCTGGGGC | p.81_82insRPSCC |  |  |
|  |  | 39305785 | 39305785 | Missense | 1 | A\|T | p.C79S |  | T(1) |
| *OR2T35* | 1 | 248801602 | 248801603 | Frame_Shift_Ins | 1 | .\|CA | p.I320fs | prostate(1) |  |
|  |  | 248801945 | 248801951 | Frame_Shift_Del | 4 | CAGCACG\|. | p.CVL203fs |  |  |
| *HAVCR1* | 5 | 156464448 | 156464448 | Intron_SNP | 1 | A\|C |  |  |  |
|  |  | 156479558 | 156479572 | In_Frame_Del | 5 | TTGGAACAGTCGTCA\|. | p.MTTVP158del | ovary(1) |  |
|  |  | 156482504 | 156482504 | Silent | 1 | A\|C | p.G29G | skin(1) |  |
| *IFI27* | 14 | 94582130 | 94582131 | In_Frame_Ins | 3 | .\|GGCCATGGC | p.33_34insMAA | breast(1) |  |
| *PCSK5* | 9 | 78683019 | 78683019 | Intron_SNP | 1 | G\|T |  |  |  |
|  |  | 78790144 | 78790153 | Frame_Shift_Del | 5 | AATGGAATGA\|. | p.NGMK667fs | lung(1) |  |
|  |  | 78790149 | 78790153 | Frame_Shift_Del | 1 | AATGA\|. | p.MK669fs |  |  |
|  |  | 78808329 | 78808330 | 3'UTR_Ins | 7 | .\|AAAA |  |  |  |
|  |  | 78808345 | 78808345 | 3'UTR_Del | 1 | A\|. |  |  |  |
| *GPRIN1* | 5 | 176025679 | 176025679 | Missense | 1 | C\|T | p.R386H |  | T(0.11) |
|  |  | 176026120 | 176026143 | In_Frame_Del | 5 | CTCAAAGACCCAGGATCCTCCTTC\|. | p.231_239RKEDPGSLR>R | lung(1) |  |
| *MRPL18* | 6 | 160211646 | 160211648 | In_Frame_Del | 3 | GTT\| | p.L10del |  |  |
| *ARSD* | X | 2832607 | 2832608 | Intron_Ins | 1 | .\|ACTAATCCC |  |  |  |
|  |  | 2832880 | 2832881 | Intron_Ins | 1 | .\|AT |  |  |  |
|  |  | 2832920 | 2832921 | Intron_Ins | 1 | .\|TA |  |  |  |
|  |  | 2833534 | 2833541 | Intron_Del | 2 | TTACGCCC\|. |  |  |  |
|  |  | 2835999 | 2836007 | In_Frame_Del | 4 | CCACGCCGG\|. | p.AGV234del |  |  |
| *MAML3* | 4 | 140811064 | 140811075 | In_Frame_Del | 4 | TGCTGCTGCTGC\|. | p.505_509QQQQQ>Q |  |  |
|  |  | 140811081 | 140811082 | In_Frame_Ins | 2 | .\|TGT | p.503_503Q>QQ |  |  |
|  |  | 140811084 | 140811084 | Silent | 3 | C\|T | p.Q502Q |  |  |
|  |  | 141073959 | 141073959 | Intron_Del | 1 | A\|. |  |  |  |
| **In AML-M2** |  |  |  |  |  |  |  |  |  |
| *CEBPA* | 19 | 33792381 | 33792382 | In_Frame_Ins | 2 | .\|CTT | p.313_314insK | haematopoietic_and_lymphoid_tissue(57) |  |
|  |  | 33792382 | 33792383 | In_Frame_Ins | 1 | .\|TTT | p.313_313K>KK | haematopoietic_and_lymphoid_tissue(62) |  |
| *EP400* | 12 | 132476120 | 132476121 | Intron_Del | 1 | TC\|. |  |  |  |
|  |  | 132547068 | 132547069 | In_Frame_Ins | 1 | .\|GCA | p.2748_2749insQ | lung(2) |  |
|  |  | 132547093 | 132547094 | In_Frame_Ins | 3 | .\|CAG | p.2728_2728Q>QQ | lung(3)\|kidney(2)\|endometrium(2)\|central_nervous_system(2) |  |
|  |  | 132547094 | 132547102 | In_Frame_Del | 1 | CAGCAGCAG\|. | p.QQQ2746del |  |  |
| **In AML-M3** |  |  |  |  |  |  |  |  |  |
| *ATXN3* | 14 | 92537353 | 92537354 | In_Frame_Ins | 1 | .\|GCTGCTGCTGCTGCT | p.306_306G>EQQQQR | lung(2) |  |
|  |  | 92537354 | 92537355 | In_Frame_Ins | 1 | .\|CTGCTGCTGCTGCTG | p.304_305insQQQQQ | lung(1) |  |
|  |  | 92562967 | 92562968 | Intron_Ins | 1 | .\|AA |  |  |  |

Abbreviation: ALT, alternative allele; Chr, chromosome; REF, reference allele

^a^Number of recurrent somatic mutations in 36 Korean AML patients.

^b^Number of reports retrieved from the COSMIC database.

^c^Functional impact score of each mutation predicted by SIFT.

**Table S3**. Results of gene ontology and KEGG pathway analyses

| **Term** | **Count** | **%** | ***p* Value** | **Fold Enrichment** | **FDR** |
| --- | --- | --- | --- | --- | --- |
| **Biological processes (BP)** | | | | | |
| GO:0007165~signal transduction^a^ | 43 | 8.85 | 0.002 | 1.63 | 0.029 |
| GO:0050890~cognition^b^ | 6 | 1.23 | 0.003 | 6.21 | 0.043 |
| **Cellular components (CC)** | | | | | |
| GO:0005886~plasma membrane^c^ | 131 | 26.95 | 3.07×10^-4^ | 1.32 | 0.004 |
| GO:0045095~keratin filament^d^ | 9 | 1.85 | 0.003 | 3.73 | 0.039 |
| **Molecular functions (MF)** | | | | | |
| GO:0097110~scaffold protein binding^e^ | 7 | 1.44 | 8.65×10^-4^ | 6.18 | 0.012 |
| **Kyoto Encyclopedia of Genes and Genomes (KEGG)** | | | | | |
| hsa04010:MAPK signaling pathway^f^ | 18 | 3.70 | 7.67×10^-4^ | 2.50 | 0.009 |
| hsa04720:Long-term potentiation^g^ | 8 | 1.65 | 0.002 | 4.28 | 0.030 |

Abbreviation: FDR, false discovery rate.

^a^*ZNF536, C3, FPR3, AKAP9, FGF13, GAST, PGR, CSNK2A1, MYD88, LILRA2, NPM1, CHRNA7, PLCB1, TYRO3, BCR, OR52B4, SIT1, MAP2K3, GABRA5, COL15A1, NR4A1, PTPRT, CNGA3, ITPR3, IRS1, FLNB, SMC3, TENM4, LILRB1, NCK2, LILRB2, PLCG1, NTS, OR3A3, RPS6KA1, RPS6KA2, IGSF1, GRN, TENM2, OR51A2, MADCAM1, RIT1, LHB*

^b^*OR52B4, SHROOM4, NF1, CHRNA7, DOPEY2, JAKMIP1*

^c^*GPRIN1, MICA, GRIK2, OR2J3, OR1E1, FGF13, OR4L1, UBQLN2, OR4C5, SLC7A6, NRCAM, LILRA1, CSNK2A1, MYD88, PRRT2, CD46, SLC9B1, CHRNA7, MTUS1, STAG2, OR7A10, FAM21C, MDGA1, MDGA2, HLA-A, ZHX2, GPR132, PTPRT, HLA-B, IRS1, SLC34A3, LILRB1, LILRB2, CD163L1, OR5H14, RASGRF2, ATP9A, OR5H15, MADCAM1, EMP3, DBN1, ARFGAP2, FCER2, IFITM3, OR2T4, CSMD3, CACNB4, KCNA5, LRIG3, KRAS, P2RY1, OR2T33, OR2T35, HLA-DPB1, OR2T34, TBC1D30, VSTM4, GABRA5, CELSR3, OR6C76, EPHA1, EPHA3, NOTCH2, GPR32, EPHA6, PLCG1, KIAA0922, NOTCH4, MTNR1B, TDG, OR51A2, TM4SF20, RIT1, CACNA1E, MARCKS, CACNA1F, SYT15, KCNH1, PCDHGA6, PCDHGA5, PCDHGA3, OR4C12, CNTNAP3, ATP8B2, DLG3, PIK3AP1, RHOD, FCGR3A, DPP6, NFX1, OR52B4, SIT1, CACNG6, ATP6V1H, SLC3A1, FLNB, TRPM1, GNAQ, ALG10B, OR4N5, ADAM12, KCNH5, USP6, SLC39A14, PARD3, C3, NPY2R, OR5D18, FPR3, RGMA, UNC79, TNFRSF1B, OR10G9, KRT5, FAT4, FAT1, FZD9, CR2, SLC12A4, HSPG2, CRIPAK, ITPR3, PLG, NRAS, OR3A3, BNC2, SVIL, TENM2, SLC18A2, ABCC5, FEZ1*

^d^*KRTAP4-9, KRTAP4-5, KRT5, KRTAP29-1, KRTAP9-1, KRTAP4-11, KRT8, KRTAP10-6, KRT84*

^e^*NCK2, KRT5, KRT8, P2RY1, CACNA1G, KCNA5, IKBKB*

^f^*MAP2K3, CACNG6, NF1, NR4A1, FGF13, CACNB4, FLNB, NRAS, KRAS, RASGRF2, RPS6KA1, RPS6KA2, CACNA1G, MOS, CACNA1E, CACNA1F, IKBKB, DUSP8*

^g^*NRAS, KRAS, EP300, RPS6KA1, GNAQ, RPS6KA2, PLCB1, ITPR3*
